# Plug-in-Gait calculation of the knee adduction moment in people with knee osteoarthritis during shod walking: comparison of two different foot marker models

**DOI:** 10.1186/s13047-017-0187-4

**Published:** 2017-02-04

**Authors:** Kade L. Paterson, Rana S. Hinman, Ben R. Metcalf, Kim L. Bennell, Tim V. Wrigley

**Affiliations:** Centre for Health, Exercise and Sports Medicine, Department of Physiotherapy, School of Health Sciences, Faculty of Medicine Dentistry & Health Sciences, The University of Melbourne, Melbourne, VIC Australia

**Keywords:** Biomechanics, Multi-segment foot model, OFM, Gait analysis, Footwear

## Abstract

**Background:**

Understanding how kinematic multi-segment foot modelling influences the utility of Plug-in-Gait calculations of the knee adduction moment (KAM) during shod walking is relevant to knee osteoarthritis (OA). Multi-segment foot markers placed on the skin through windows cut in to the shoe provide a more accurate representation of foot mechanics than the traditional marker set used by Plug-in-Gait, which uses fewer markers, placed on the shoe itself. We aimed to investigate whether Plug-in-Gait calculation of the KAM differed when using a kinematic multi-segment foot model compared to the traditional Plug-in-Gait marker set.

**Methods:**

Twenty people with medial knee OA underwent gait analysis in two test conditions: i) Plug-in-Gait model with its two standard foot markers placed on the shoes and; ii) Plug-in-Gait with the heel marker virtualised from a modified-Oxford Foot Model where 8 ft markers were placed on the skin through windows cut in shoe uppers. Outcomes were the peak KAM, KAM impulse and other knee kinetic and kinematic variables.

**Results:**

There were no differences (*P* > 0.05) in any gait variables between conditions. Excellent agreement was found for all outcome variables, with high correlations (*r *> 0.88-0.99, *P* < 0.001), narrow limits of agreement and no proportional bias (*R* ^2^ = 0.03–0.14, *P* > 0.05). The mean difference and 95% confidence intervals for peak KAM were also within the minimal detectable change range demonstrating equivalence.

**Conclusions:**

Plug-in-Gait calculations of the KAM are not altered when using a kinematic multi-segment foot marker model with skin markers placed through windows cut in to the shoe, instead of the traditional marker set placed on top of shoes. Researchers may be confident that applying either foot model does not change the calculation of the KAM using Plug-in-Gait.

**Electronic supplementary material:**

The online version of this article (doi:10.1186/s13047-017-0187-4) contains supplementary material, which is available to authorized users.

## Background

Knee osteoarthritis (OA) is a common condition that causes significant pain and disability. It affects the medial compartment of the knee more often than the lateral [1], which is likely due to increased loading in the medial compared to the lateral tibiofemoral joint during walking [2]. Due to difficulties with direct in vivo measurement of joint loading, dynamic knee load distribution is commonly inferred using the knee adduction moment (KAM) from three dimensional gait analysis. Research has shown that the KAM is higher in people with medial knee OA compared to healthy controls [2], and is significantly associated with both knee pain [3] and structural disease progression [4–6]. A higher KAM at baseline has also been shown to significantly increase the risk of progression to a total knee replacement over a 5–8 year period [7]. For these reasons, reducing the KAM through conservative biomechanical interventions has become a major research focus in global efforts to improve management of knee OA.

The foot is a common target for biomechanical interventions designed to reduce the KAM because foot mechanics can influence knee joint loading [8]. For example in people with knee OA, laterally-wedged insoles placed inside footwear reduce the KAM by an average of 5–12% [9–12], whilst other research shows the KAM is significantly decreased when walking in specially modified shoes with soles of variable stiffness [13–15] or flat flexible soles [16–18]. However, individual response to these foot-based interventions is somewhat variable, with studies showing the KAM is actually increased in up to 30% of people wearing lateral wedges [9, 11, 19] and in approximately 15% of people wearing modified shoes with variable stiffness soles [13, 20]. This may help to explain why large clinical trials of lateral wedges [21] and variable stiffness shoes [22] have failed to show clinical superiority of these interventions over control conditions. Thus, there is growing research, and much interest in concurrent measurement of multi-segmental foot kinematics and the KAM during shod walking in people with knee OA [23].

The most commonly used biomechanical model - the variously-named ‘conventional gait model’ or Plug-in-Gait model (amongst others) - does not enable accurate measurement of multi-segment foot kinematics because it typically includes only two markers on the foot. Reducing the foot to this basic model is overly simplistic for some foot motions. A recent study reported that ankle motion concurrently calculated using Plug-in-Gait and a commonly used kinematic multi-segment foot model, the Oxford Foot Model (OFM) [24], was not only significantly different throughout the entire gait cycle, but one reported the simple whole-foot model to be dorsiflexed at heel strike (Plug-in-Gait), while the other reported the more accurately-modelled rearfoot to be plantarflexed (OFM) [25]. This suggests that researchers should use a multi-segment foot model for measuring foot kinematics.

To investigate foot kinematics during shod walking, the use of multi-segment foot models requires the placement of markers on the skin through ‘windows’ cut in to the shoes. In locations where this could excessively compromise the structural integrity of the shoe, marker wands and ‘virtual’ calculated markers may also be required. However, it is unknown whether creating virtual traditional foot model markers influences the calculation of KAM using Plug-in-Gait. In addition, potential differences in Plug-in-Gait calculations of the KAM may arise from shoe (and thus marker) movement relative to the foot when using the traditional model, and/or from potential for skin artefact, wand oscillation at heel contact and/or altered foot and/or marker movement due to loss of shoe integrity from cutting of ‘windows’ for skin-mounted markers when using the kinematic multi-segment foot model in a modified shoe. Any or all of these variables are biomechanically plausible mechanisms through which shank calculations, and therefore the KAM, may be affected through ‘bottom-up’ inverse dynamics. Thus, if further studies evaluating the relationships between foot biomechanics, the KAM and clinical outcomes from foot-based treatment interventions are to be conducted, it is essential to first establish that concurrent measurement of in-shoe foot biomechanics does not alter the KAM in people with knee OA. The aim of this study was to investigate whether Plug-in-Gait calculation of the KAM differed when using an in-shoe kinematic multi-segment foot model compared to the traditional Plug-in-Gait marker set.

## Methods

### Participants

Twenty participants were recruited from a clinical trial investigating the effects of unloading footwear in people with knee OA [26]. For the clinical trial, participants were recruited from the community using advertisements, social media, media campaigns and from our existing network of volunteers. Participants were aged 50 or over, reported knee pain on most days of the previous month, had definite radiographic evidence of knee OA (defined as Kellgren & Lawrence (KL) grade ≥ 2 [27]); demonstrated medial tibiofemoral compartment OA on x-ray (defined as ≥ grade 1 medial osteophytes and ≥ grade 1 medial joint space narrowing [28]) and reported a minimum average pain score of 4 in the past week on an 11-point numerical rating scale (terminal descriptors of ‘no pain’ and ‘worst pain possible’). Major exclusion criteria were predominant lateral tibiofemoral OA or other knee pathology likely to be causing knee pain; intra-articular cortisone injection in the past 3 months, knee surgery in the previous 6 months or planned knee surgery in the subsequent 6 months; other muscular, joint or neurological condition with the potential to affect lower limb function; current or previous (last 6 months) use of a shoe insert, knee or ankle brace; unable to walk unaided; body mass index ≥ 36 kg/m^2^ or any ankle or foot pain/pathology.

For this nested study, we recruited participants from the control group after they had completed their final assessment for the clinical trial at 6 months. All procedures were approved by the University of Melbourne Human Research Ethics Committee (ID 1239045) and all participants provided informed consent.

### Radiographs

Participants underwent weightbearing semi-flexed radiographs of both knees. Radiographs were evaluated to determine the KL grade [27], medial and lateral tibial and femoral osteophytes, and medial and lateral tibiofemoral joint space narrowing [28]. The KL grade rates OA radiographic disease severity from Grade 0 (no sign of knee OA) to Grade 4 (severe OA) [27]. Severity of tibial and femoral osteophytes and tibiofemoral joint space narrowing in the medial and lateral compartments were graded from 0 (normal) to 3 (severe change) [28].

Anatomic knee alignment was also determined from the radiographs. This was measured as the angle between medial-lateral bisections of the femur and tibia, made 10 cm above or below the tibiofemoral joint line, and passing through the midpoint of the tibial spines [29].

### Footwear conditions and foot marker placement

Participants underwent 3D gait analysis in neutral cushioned athletic walking shoes (ASICS Odyssey) under two different conditions presented in random order: i) Plug-in-Gait model, wearing intact shoes and the standard two “foot” markers placed on the shoe and; ii) Plug-in-Gait plus the OFM marker set, with shoes that have six small windows cut into the uppers to permit placement of 8 retro-reflective markers directly on the skin.

**Fig. 1:**
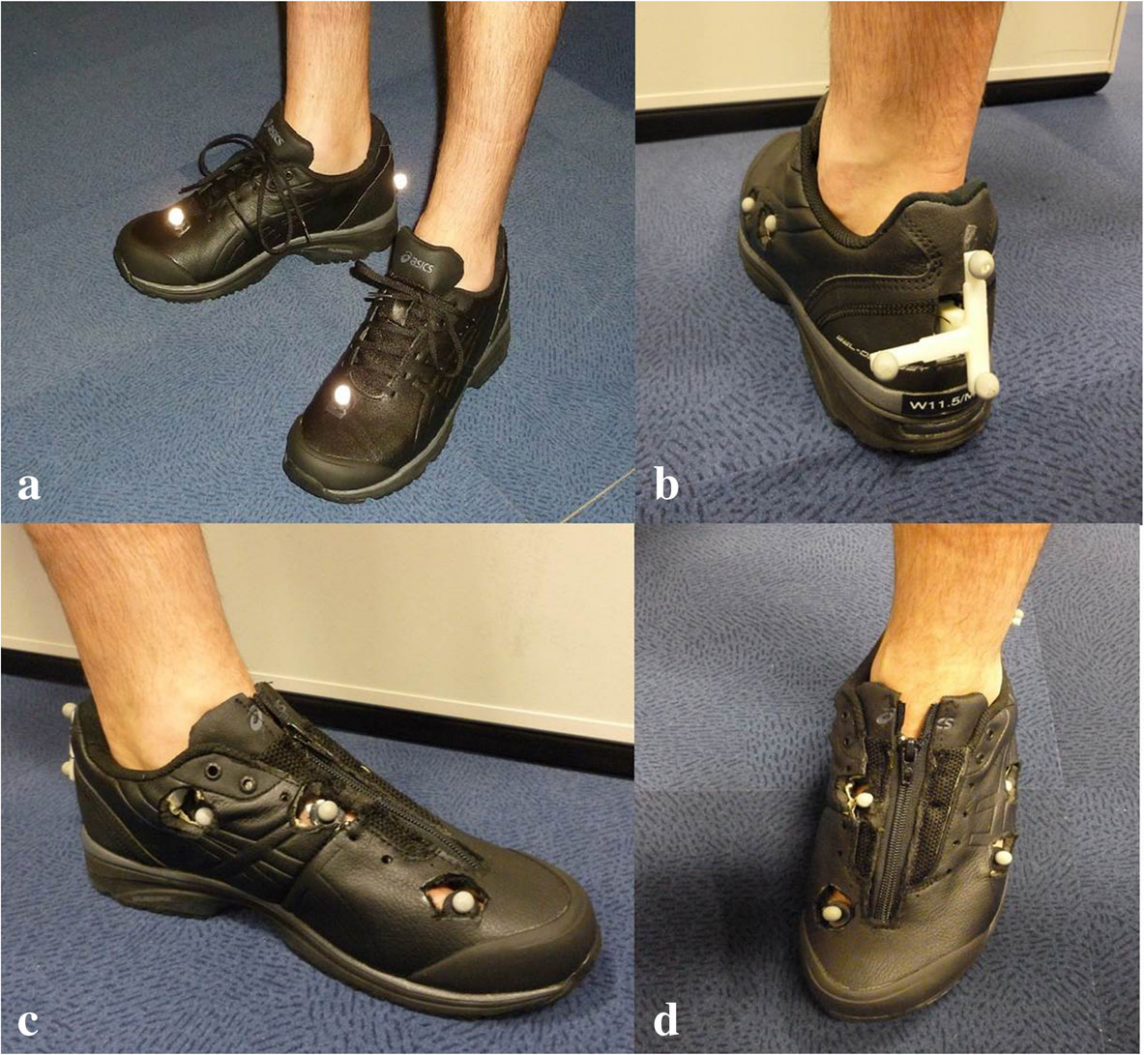
**a** Plug-in-Gait HEE and TOE markers in the intact shoe marker set, (**b**) posterior view of our Plug-in-Gait plus modified-OFM cut shoe showing the rearfoot wand, (**c**) medial view of the foot model and shoe, and (**d**) superior view of our foot model showing the zip

For the Plug-in-Gait condition, 17 retro-reflective markers were adhered to lower limb anatomical landmarks on the skin and shoe using the standard Plug-in-Gait (Vicon, UK) marker set. On the shoe, this model included markers on the dorsal second metatarsal head (‘TOE’ marker) and posterior calcaneus (‘HEE’ marker) (Fig. 1a). Further details regarding Plug-in-Gait and the kinematic and kinetic calculations used by the model can be found elsewhere [30, 31].

A similar marker set was used in the Plug-in-Gait plus modified-OFM condition however the two shoe-mounted foot markers were replaced with eight markers directly adhered to the skin through the six shoe windows (Fig. 1b and c). Window sizes were a maximum of 1.7 x 2.5 cm [32]. The tongue and laces of the shoe were replaced with a zip to permit donning and doffing without the need to remove or replace foot markers (Fig. 1d).

Our foot marker set for the OFM was based on the standard Plug-in-Gait and OFM marker set [24] with two minor modifications. Firstly, the OFM requires three posterior calcaneal markers; however cutting windows in the rear of the shoe’s heel counter for visualisation of these markers violated the minimum window size for shoe integrity [32]. Therefore, we used a single, 3D-printed, rearfoot triad/wand with three markers fixed to a small, curved plastic rearfoot plate adhered centrally on the skin, with its vertical axis aligned with a bisection of the calcaneus (Fig. 1b) [33]. In addition to allowing clear visualisation of the calcaneal markers, this also permitted the wand to be attached through the rearfoot shoe window with the rearfoot plate already fixed to the posterior calcaneus (prior to donning the shoe). The triad coordinate system was used to reconstruct a virtual Plug-in-Gait ‘HEE’ heel marker in a similar manner to the standard Plug-in-Gait plus OFM marker set. Briefly, the triad markers have a known, fixed coordinate relationship to two small holes on the triad base plate on the rearfoot, which are aligned directly over the drawn rearfoot bisection line when the base plate is attached. Thus virtual marker locations such as the HEE and proximal calcaneal marker can be determined on the rearfoot bisection directly from the triad coordinate frame, as they would be if placed directly on the skin. For example, the LHEE is fixed at the distal hole location of (40,0,-35) in the triad coordinate frame. The approach is similar to that in Telfer et al [33]. The Plug-in-Gait ‘TOE’ marker was placed on the skin through a hole in the shoe. An addition to the OFM markers was a marker placed on the navicular tuberosity to assess dynamic navicular movement within the shoe for our larger trial (this marker was not used in the calculation of any outcomes for this study). The operation of our modified OFM model is not described here, as no OFM-derived data is included here. For generalizability of our results, note that the Plug-in-Gait plus OFM calculations are done with the standard Vicon OFM pipeline, as the modified model yields the same final marker locations as the standard Plug-in-Gait and OFM.

### Gait analysis

Biomechanical data were collected while participants walked at self-selected normal speed in the two shoe test conditions presented in random order. Walking speed was monitored using two photoelectric beams 4 m apart, and verbal feedback was used to ensure that speed was maintained within 5% from the average walking speed of the condition that was completed first. Kinematic data were collected using a 12-camera Vicon MX motion analysis system (Vicon, Oxford, UK) sampling at 120 Hz, and ground reaction force data were collected using two floor mounted force plates (Advanced Medical Technology Inc., Watertown, MA, USA) at 1,200 Hz. Averages of discrete data from six successful walking trials was used for all outcomes measures. External joint moments were calculated in Vicon Nexus 1.85 (Vicon, Oxford, UK) from the six trials using standard “bottom-up” inverse dynamics using Plug-in-Gait markers only for both conditions (Vicon Plug-in-Gait v2, Oxford, UK). The ankle joint centre was calculated using the traditional HEE marker placed externally on the shoe for the traditional Plug-in-Gait condition, whereas the virtual HEE marker (created using the three wand markers adhered directly to the skin through a window cut in the shoe heel counter) was used for the Plug-in-Gait plus modified-OFM condition. OFM does not calculate moments; it is a kinematics-only model. Knee moments were expressed in the distal (shank) coordinate system and normalised for body weight times height, expressed as Nm/BWxHt%. The primary outcome variables for this study were the first and second peak KAM and the KAM impulse. KAM impulse (the positive area under the KAM-time curve) was included in addition to the KAM as it reflects the mean KAM and its duration during stance. Secondary outcome variables were other knee kinematics and kinetics in the sagittal, frontal and transverse planes. Spatiotemporal gait data were reported for descriptive purposes. As the focus of this study was to investigate whether Plug-in-Gait calculation of the KAM differed when using a kinematic multi-segment foot model compared to the traditional Plug-in-Gait marker set, foot-related variables calculated by the Vicon Nexus OFM plugin are not relevant and are thus not reported here.

### Statistical analysis

Statistical analyses were performed using the Statistical Package for the Social Sciences (version 22.0, IBM, Armonk, NY, USA) with an alpha level of 0.05. Data were described as mean (standard deviation (SD)). To examine whether the primary and secondary knee variables differed across the two test conditions, mean differences (95% confidence intervals (CI)) between the models were calculated and repeated measures multivariate analyses of variance (MANOVA) performed. Where appropriate, Bonferroni post hoc tests were performed to correct for multiple pairwise comparisons. Separate MANOVAs were conducted for knee kinetic and kinematic data. Next, associations between data from each condition was assessed using Pearson’s correlations, and r values were interpreted as poor (<0.40), modest (0.40–0.74) or excellent (>0.75) [34]. Agreement between outcomes was examined using Bland Altman plots and 95% limits of agreement (LOA). This method plots the difference between test conditions and the average of the two results. The plots were visually inspected to evaluate the distribution of data points within ±1.96SD of the mean difference, and proportional bias was examined using linear regression to assess whether there was a linear trend in data falling above or below the mean difference. Finally, we examined equivalence [35] between peak KAM calculated using the two foot marker models by determining whether the mean difference and associated confidence intervals fell wholly within minimal detectable change value range of ±0.101 Nm/BWxHt% [36].

## Results

Participant characteristics are presented in Table 1. Nearly two thirds (65%) of the sample were women. On average, participants were in their early sixties and had experienced OA symptoms for over 8 years. Participants were generally overweight and reported moderate pain. Radiographic severity was mostly moderate to severe, with three quarters (75%) having a KL grade 3 or 4.

**Table 1:** Descriptive characteristics of the group. Data presented as mean (SD) unless indicated otherwise

Characteristics	*n* = 20
Female gender, n (%)	13 (65)
Age (years)	63 (7)
Duration of OA (years)	8 (8)
Height (m)	1.65 (0.09)
Mass (kg)	78.3 (14.0)
Body mass index (kg/m^2^)	28.7 (3.7)
Average pain in the last week on 11-point rating scale	6 (2)
Radiographic severity (KL grade), n (%)	
Grade 2	5 (25)
Grade 3	9 (45)
Grade 4	6 (30)
Medial femoral osteophytes, n (%)	
Grade 0	2 (10)
Grade 1	4 (20)
Grade 2	8 (40)
Grade 3	6 (30)
Medial tibial osteophytes, n (%)	
Grade 0	1 (5)
Grade 1	9 (45)
Grade 2	8 (40)
Grade 3	2 (10)
Lateral femoral osteophytes, n (%)	
Grade 0	10 (50)
Grade 1	6 (30)
Grade 2	2 (10)
Grade 3	2 (10)
Lateral tibial osteophytes, n (%)	
Grade 0	5 (25)
Grade 1	9 (45)
Grade 2	5 (25)
Grade 3	1 (5)
Medial tibiofemoral narrowing, n (%)	
Grade 0	0 (0)
Grade 1	4 (20)
Grade 2	8 (40)
Grade 3	8 (40)
Lateral tibiofemoral narrowing, n (%)	
Grade 0	17 (85)
Grade 1	3 (15)
Grade 2	0 (0)
Grade 3	0 (0)
Knee Alignment (°)	177.3 (4.2)

*OA* osteoarthritis, *KL* Kellgren Lawrence

Descriptive statistics obtained across test conditions are presented in Table 2, and mean differences, agreement and correlations between conditions are presented in Table 3. Knee kinetic and kinematic gait variables were similar across both conditions, with very small mean differences and narrow associated confidence intervals. Separate MANOVAs conducted for the kinetic and kinematic data demonstrated that these differences were not significant (*P* > 0.05). Likewise, excellent agreement between the two conditions was also found, with narrow limits of agreement and excellent Pearson’s correlation coefficients (*r* = 0.84–0.99).

**Table 2:** Descriptive statistics for the gait variables in each test condition. Data are reported as mean (SD)

Gait variables	PiG plus-OFM cut shoe	PiG intact shoe
*Knee kinetics*		
First peak KAM (Nm/BWxHt%)	4.99 (1.07)	4.93 (1.00)
Second peak KAM (Nm/BWxHt%)	2.80 (0.93)	2.82 (0.99)
KAM impulse (Nm.s/BWxHt%)	1.59 (0.41)	1.59 (0.39)
Peak knee flexion moment (Nm/BWxHt%)	3.02 (2.00)	3.26 (1.82)
*Knee kinematics*		
Peak knee flexion in loading response (°)	18.52 (7.30)	18.41 (6.77)
Peak knee extension in stance (°)	5.38 (5.89)	5.44 (5.74)
Peak knee adduction in stance (°)	10.31 (5.83)	10.64 (5.61)
Peak internal rotation in stance (°)	11.69 (5.88)	11.26 (5.73)
*Spatiotemporal data*		
Stance duration (secs)	0.68 (0.06)	0.67 (0.06)
Stride length (m)	1.46 (0.16)	1.46 (0.15)
Cadence (strides/min)	56.55 (4.54)	56.80 (4.40)
Velocity (m/s)	1.38 (0.20)	1.38 (0.19)
Base of support (m)	0.13 (0.02)	0.13 (0.02)

*KAM* knee adduction moment, *PiG* Plug-in-Gait

**Table 3:** Mean difference (95% confidence intervals (CI)), 95% limits of agreement (LoA) and Pearson’s correlation coefficients between gait variables in each condition

Gait variables	Mean difference (95% CI)	*P* value	95% LoA	r
*Knee kinetics*				
First peak KAM (Nm/BWxHt%)	-0.06 (-0.18 to 0.07)	0.34	0.46 to -0. 57	0.97^**^
Second peak KAM (Nm/BWxHt%)	0.02 (-0.06 to 0.10)	0.61	0.35 to -0.31	0.98^**^
KAM impulse (Nm.s/BWxHt%)	-0.00 (-0.04 to 0.03)	0.94	0.14 to -0.14	0.99^**^
Peak knee flexion moment (Nm/BWxHt%)	0.23 (-0.02 to 0.48)	0.07	1.27 to -0.81	0.97^**^
*Knee kinematics*				
Peak knee flexion in loading response (°)	-0.12 (-1.03 to 0.79)	0.79	3.69 to -3.92	0.97^**^
Peak knee extension in stance (°)	0.06 (-0.71 to 0.84)	0.87	3.31 to -3.18	0.96^**^
Peak knee adduction in stance (°)	0.37 (-0.63 to 1.36)	0.45	4.54 to -3.81	0.93^**^
Peak internal rotation in stance (°)	-0.47 (-1.82 to 0.89)	0.47	5.18 to -6.12	0.89^**^

*KAM* knee adduction moment

^**^ *P* < 0.001

**Fig. 2:**
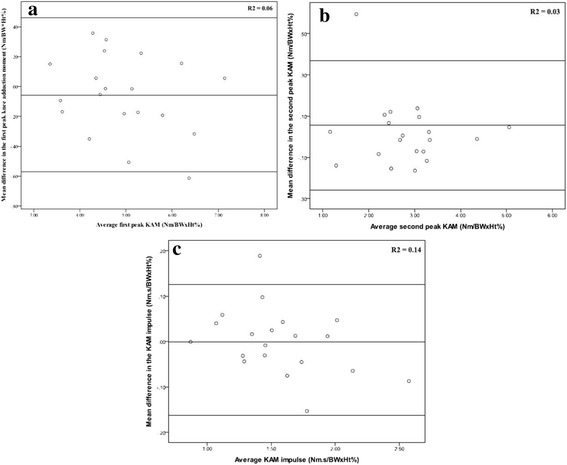
Bland Altman plots and the 95% limits of agreement for the (**a**) first peak knee adduction moment, (**b**) second peak knee adduction moment, and (**c**) the knee adduction moment impulse between conditions. The mean difference between conditions is shown on the Y-axis and the standard deviation of the difference is shown on the X-axis. Regression coefficient of proportional bias in data falling above or below the mean difference is shown inset

Inspection of the Bland Altman plots for the primary outcomes demonstrates the majority of data points were distributed closely around the mean difference (Fig. 2). Linear regression showed there were no linear trends in the bias of individual data points relative to the mean difference (*R* ^2^ = 0.03–0.14, *P* > 0.05). Similar outcomes regarding the Bland Altman plots and regression models were also found for all secondary variables (see Additional file 1). Finally, the mean difference in peak KAM, and the associated 95% CI, were within the minimal detectable change range of ±0.101 Nm/BWxHt% demonstrating equivalence.

## Discussion

This study investigated whether Plug-in-Gait calculation of the KAM differed when using a kinematic multi-segment foot model compared to the traditional Plug-in-Gait marker set. The results demonstrated similar outcomes between the two conditions, and statistical equivalence for peak KAM, with the mean difference and 95% CIs lying wholly within the minimal detectable change range [36]. Our findings provide useful information for researchers measuring the KAM with Plug-in-Gait in people with knee OA. More simplistic traditional model with markers placed externally on the shoe yields similar KAM calculations to the more complex kinematic multi-segment foot marker model with skin-mounted markers placed through windows in the shoe.

Researchers focussed on concurrently investigating foot kinematics and knee moments can have confidence that applying kinematic multi-segment foot models (similar to that used in this study) to virtualise foot markers during shod walking in modified shoes will not impact Plug-in-Gait calculations of the KAM or other knee variables. Likewise, researchers who are only interested in the KAM and do not wish to investigate foot kinematics may instead use the traditional Plug-in-Gait marker set to calculate knee moments. As this involves foot markers placed on the outside of the shoe, there are advantages in that custom-modification of shoes to create windows for skin-mounted marker placement is not required, and that participants can be tested in their own footwear.

A potential limitation of this research may be the use of the rearfoot wand to re-create a virtual Plug-in-Gait posterior calcaneal marker, rather than the original OFM heel markers. This was done because we wanted to investigate whether the use of a kinematic multi-segment foot model, with multiple rearfoot markers placed on the skin through windows cut in to the shoe to calculate virtual Plug-in-Gait heel markers, altered important knee OA gait variables. For example, our model was a modified version of the OFM, which originally included markers on the medial and lateral calcaneus, as well as two posterior calcaneal markers [24]. However, our pilot testing showed that the shoe modifications required to visualise these markers resulted in hole sizes that would substantially violate the structural integrity of the shoe [32]. Therefore, we used the calcaneal triad/wand, attached to a small base plate that was adhered over a bisection of the calcaneus, similar to that employed by Telfer and colleagues [33]. In addition to minimising disruption to the shoe’s upper, this also facilitated donning and doffing of the footwear thus improving the feasibility of this method. Furthermore, the fact that the use of this wand to create a virtual heel marker did not affect knee biomechanics improves the generalizability of our findings given other kinematic multi-segment foot models have been used to create virtual foot markers [33, 37]. The use of only one shoe style may be a limitation of our study given different footwear styles have different effects on the KAM [18], and cutting six shoe windows may impact the integrity of some shoe styles more than others. However the style of shoe used in our study (ASICS Odyssey) is typical of everyday walking shoes worn by many people with knee OA [38], and the hole size we used for marker visualisation was based on the recommendation for this specific shoe style [32]. Our two foot modelling conditions differed with regard to a number of aspects (windows or no windows in shoes; virtualised, skin- or shoe-mounted markers), thus it is not possible to determine the individual effect of each variation on the KAM. Future studies may wish to investigate the impacts of these variations individually on knee biomechanics. Finally, it is not clear whether our findings can be generalised beyond the OFM and the Plug-in-Gait marker models. We chose to compare knee biomechanics calculated using these marker models as they are some of the most common biomechanical models used in knee OA. However, future research may consider investigating whether similar outcomes are found when calculating knee biomechanics using other lower limb and foot biomechanical models.

## Conclusions

This study showed that Plug-in-Gait calculations of the KAM are not altered when using a kinematic multi-segment foot marker model with skin markers placed through windows cut in to the shoe, instead of the traditional marker set placed on top of shoes. Given the importance of the KAM in knee OA pathogenesis, researchers may be confident that applying either foot model does not change the calculation of the KAM using Plug-in-Gait.

## Additional file

Additional file 1: Figure S1. Description: Multiple figures - supplementary limits of agreement plots for secondary outcome measures. Bland Altman plots and the 95% limits of agreement for the (A) peak knee flexion moment, (B) peak knee flexion in loading response, (C) peak knee extension in stance, (D) peak knee adduction in stance, (E) peak internal rotation in stance, (F) stance duration, (G) stride length, (H) cadence, (I) velocity and (J) base of support. The mean difference between conditions is shown on the Y-axis and the standard deviation of the difference is shown on the X-axis. Regression coefficient of proportional bias in data falling above or below the mean difference is shown inset. (ZIP 221 kb)

## Abbreviations

CI: Confidence intervals
KAM: Knee adduction moment
KL: Kellgren & Lawrence
LOA: Limits of agreement
MANOVA: Multivariate analyses of variance
OA: Osteoarthritis
ORM: Oxford Foot Model
