# Supplementary figures and images for: Plug-in-Gait calculation of the knee adduction moment in people with knee osteoarthritis during shod walking: comparison of two different foot marker models
Source: J Foot Ankle Res. 2017 Feb 4;10:8. doi: 10.1186/s13047-017-0187-4 (PMC5292150; doi:10.1186/s13047-017-0187-4)

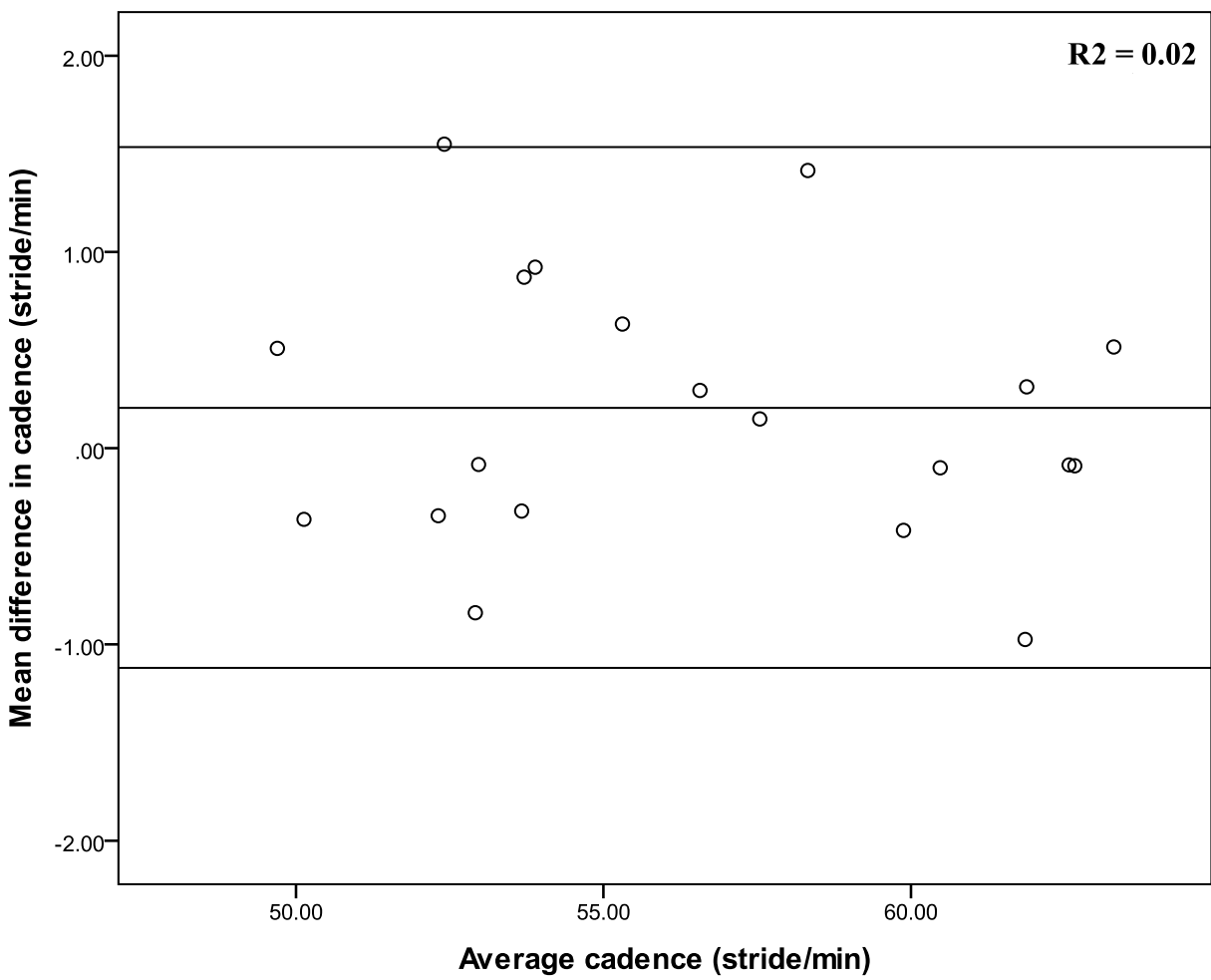

Supplement: Additional file 1: Figure S1. — Description: Multiple figures - supplementary limits of agreement plots for secondary outcome measures. Bland Altman plots and the 95% limits of agreement for the (A) peak knee flexion moment, (B) peak knee flexion in loading response, (C) peak knee extension in stance, (D) peak knee adduction in stance, (E) peak internal rotation in stance, (F) stance duration, (G) stride length, (H) cadence, (I) velocity and (J) base of support. The mean difference between conditions is shown on the Y-axis and the standard deviation of the difference is shown on the X-axis. Regression coefficient of proportional bias in data falling above or below the mean difference is shown inset. (ZIP 221 kb) [file 13047_2017_187_MOESM1_ESM.zip › Supp fig H - CadenceR3.pdf]

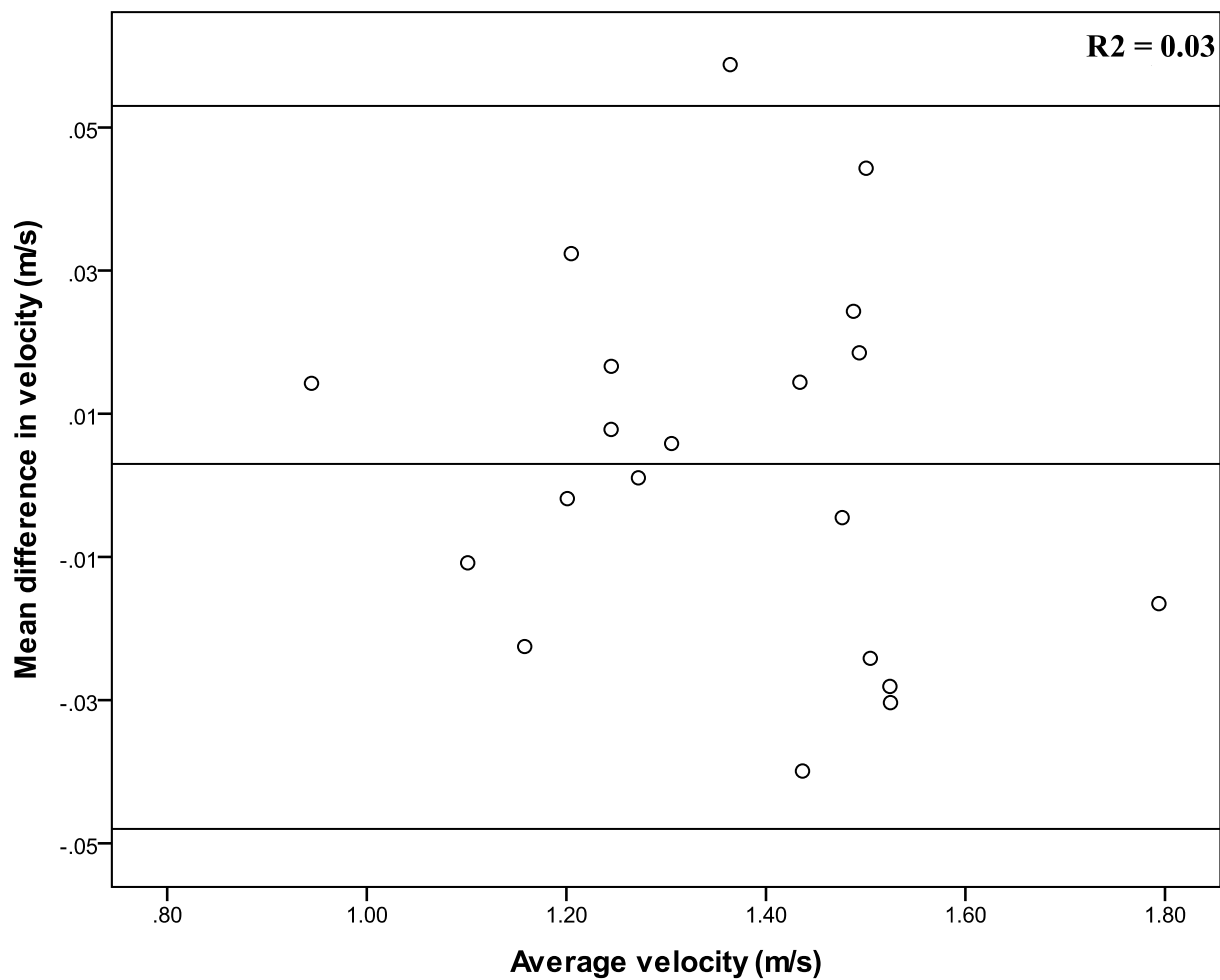

Supplement: Additional file 1: Figure S1. — Description: Multiple figures - supplementary limits of agreement plots for secondary outcome measures. Bland Altman plots and the 95% limits of agreement for the (A) peak knee flexion moment, (B) peak knee flexion in loading response, (C) peak knee extension in stance, (D) peak knee adduction in stance, (E) peak internal rotation in stance, (F) stance duration, (G) stride length, (H) cadence, (I) velocity and (J) base of support. The mean difference between conditions is shown on the Y-axis and the standard deviation of the difference is shown on the X-axis. Regression coefficient of proportional bias in data falling above or below the mean difference is shown inset. (ZIP 221 kb) [file 13047_2017_187_MOESM1_ESM.zip › Supp fig I - velR3.pdf]

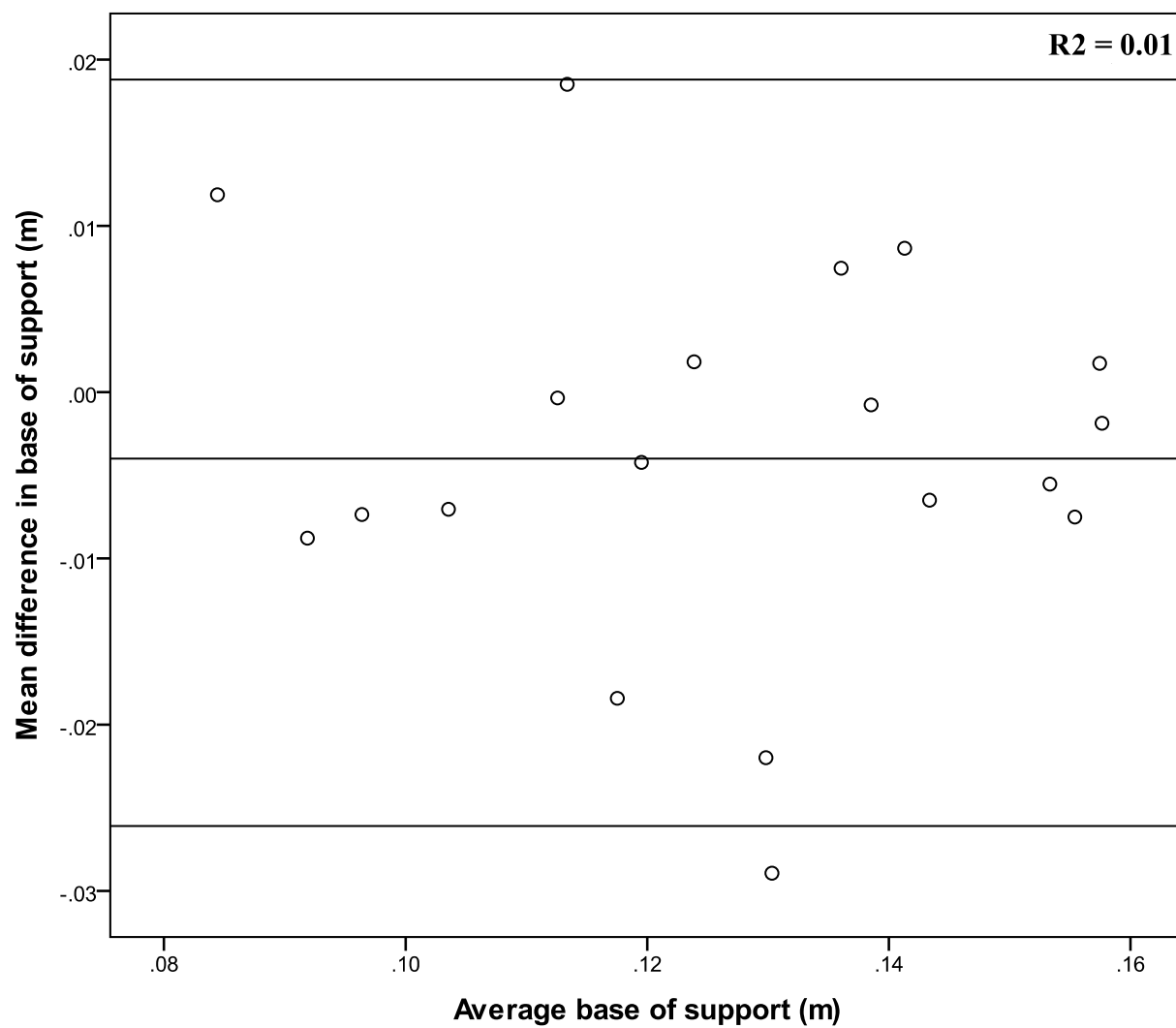

Supplement: Additional file 1: Figure S1. — Description: Multiple figures - supplementary limits of agreement plots for secondary outcome measures. Bland Altman plots and the 95% limits of agreement for the (A) peak knee flexion moment, (B) peak knee flexion in loading response, (C) peak knee extension in stance, (D) peak knee adduction in stance, (E) peak internal rotation in stance, (F) stance duration, (G) stride length, (H) cadence, (I) velocity and (J) base of support. The mean difference between conditions is shown on the Y-axis and the standard deviation of the difference is shown on the X-axis. Regression coefficient of proportional bias in data falling above or below the mean difference is shown inset. (ZIP 221 kb) [file 13047_2017_187_MOESM1_ESM.zip › Supp fig J - BoSR3.pdf]

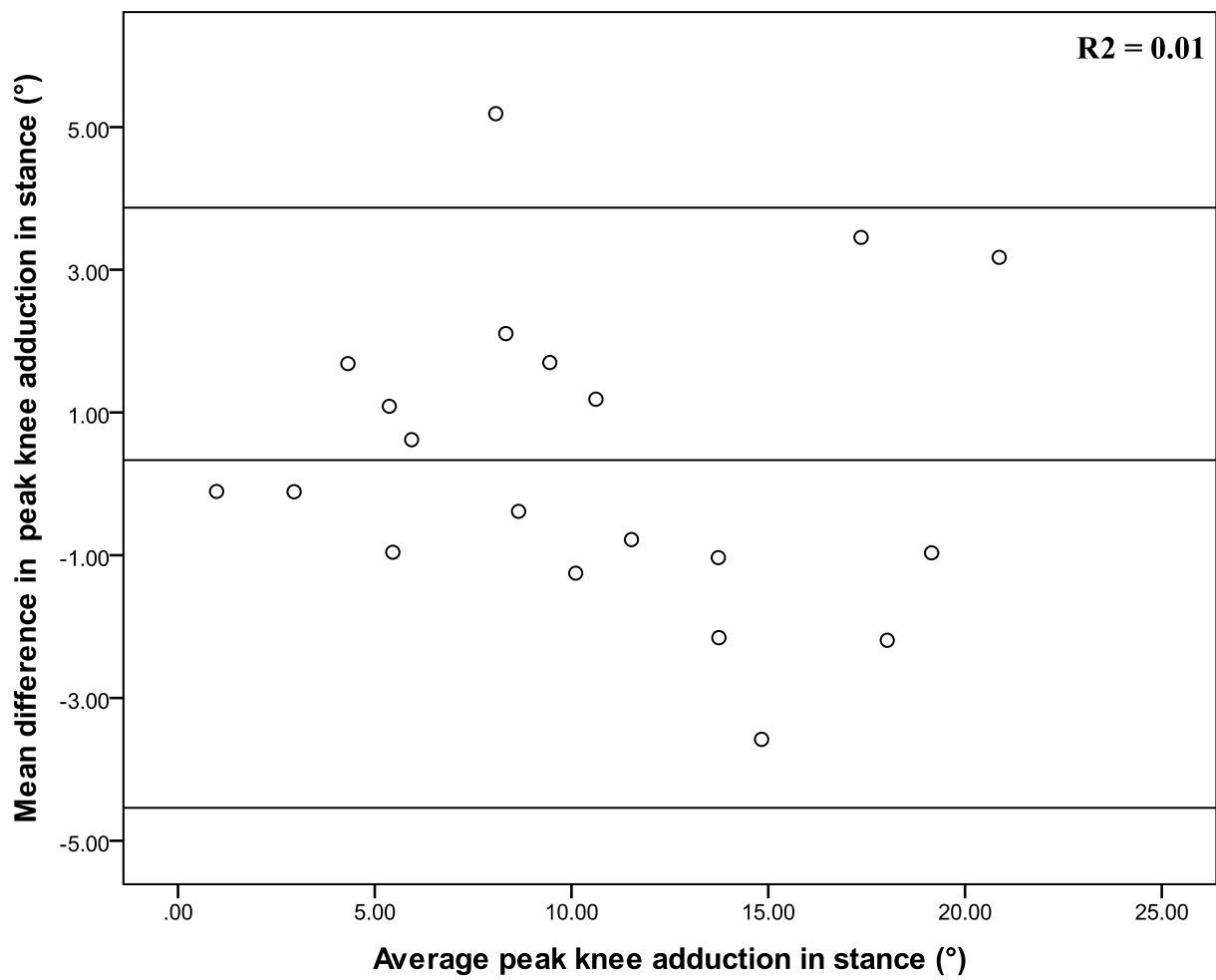

Supplement: Additional file 1: Figure S1. — Description: Multiple figures - supplementary limits of agreement plots for secondary outcome measures. Bland Altman plots and the 95% limits of agreement for the (A) peak knee flexion moment, (B) peak knee flexion in loading response, (C) peak knee extension in stance, (D) peak knee adduction in stance, (E) peak internal rotation in stance, (F) stance duration, (G) stride length, (H) cadence, (I) velocity and (J) base of support. The mean difference between conditions is shown on the Y-axis and the standard deviation of the difference is shown on the X-axis. Regression coefficient of proportional bias in data falling above or below the mean difference is shown inset. (ZIP 221 kb) [file 13047_2017_187_MOESM1_ESM.zip › Supp fig D - KAddR3.pdf]

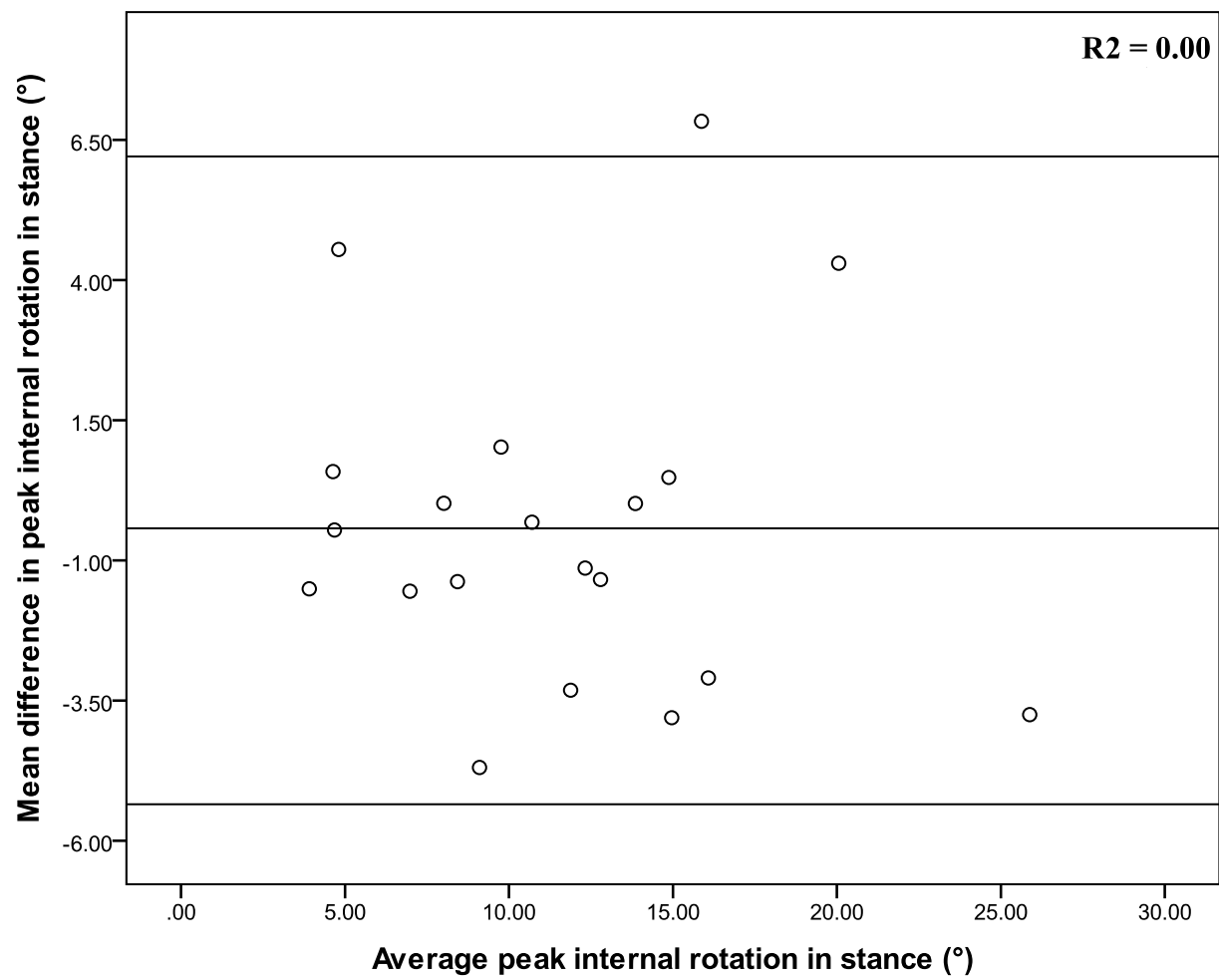

Supplement: Additional file 1: Figure S1. — Description: Multiple figures - supplementary limits of agreement plots for secondary outcome measures. Bland Altman plots and the 95% limits of agreement for the (A) peak knee flexion moment, (B) peak knee flexion in loading response, (C) peak knee extension in stance, (D) peak knee adduction in stance, (E) peak internal rotation in stance, (F) stance duration, (G) stride length, (H) cadence, (I) velocity and (J) base of support. The mean difference between conditions is shown on the Y-axis and the standard deviation of the difference is shown on the X-axis. Regression coefficient of proportional bias in data falling above or below the mean difference is shown inset. (ZIP 221 kb) [file 13047_2017_187_MOESM1_ESM.zip › Supp fig E - KIRR3.pdf]

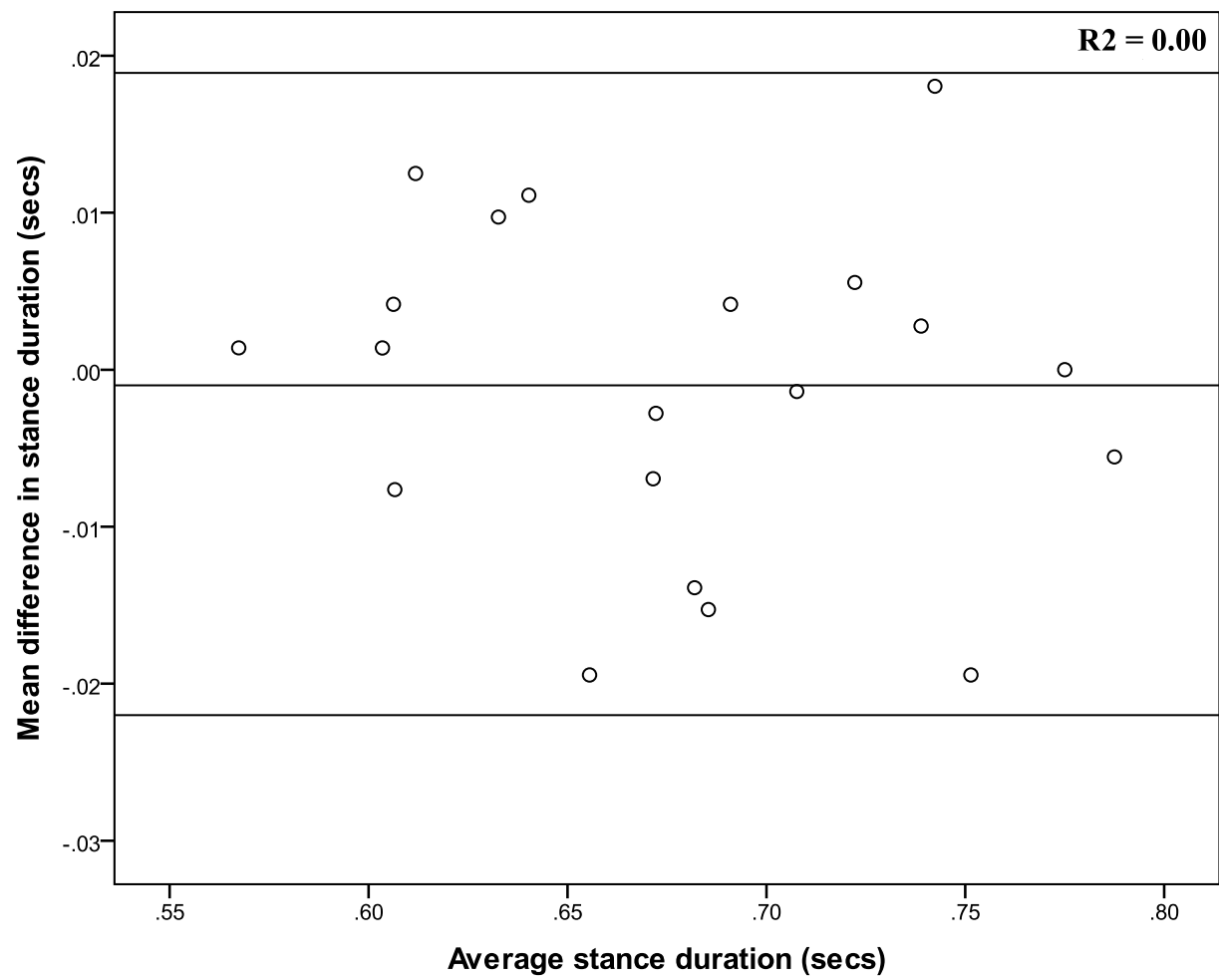

Supplement: Additional file 1: Figure S1. — Description: Multiple figures - supplementary limits of agreement plots for secondary outcome measures. Bland Altman plots and the 95% limits of agreement for the (A) peak knee flexion moment, (B) peak knee flexion in loading response, (C) peak knee extension in stance, (D) peak knee adduction in stance, (E) peak internal rotation in stance, (F) stance duration, (G) stride length, (H) cadence, (I) velocity and (J) base of support. The mean difference between conditions is shown on the Y-axis and the standard deviation of the difference is shown on the X-axis. Regression coefficient of proportional bias in data falling above or below the mean difference is shown inset. (ZIP 221 kb) [file 13047_2017_187_MOESM1_ESM.zip › Supp fig F - SDR3.pdf]

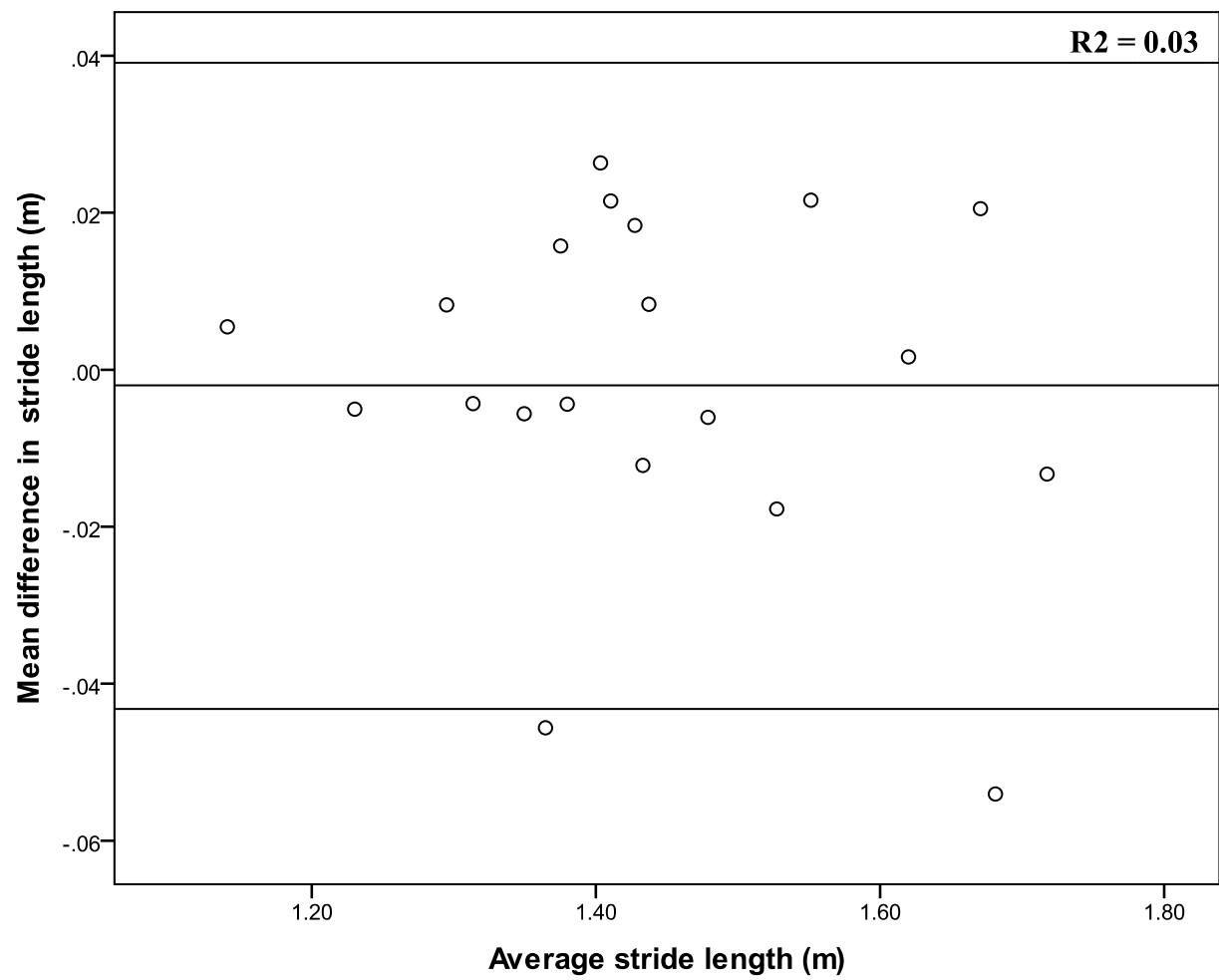

Supplement: Additional file 1: Figure S1. — Description: Multiple figures - supplementary limits of agreement plots for secondary outcome measures. Bland Altman plots and the 95% limits of agreement for the (A) peak knee flexion moment, (B) peak knee flexion in loading response, (C) peak knee extension in stance, (D) peak knee adduction in stance, (E) peak internal rotation in stance, (F) stance duration, (G) stride length, (H) cadence, (I) velocity and (J) base of support. The mean difference between conditions is shown on the Y-axis and the standard deviation of the difference is shown on the X-axis. Regression coefficient of proportional bias in data falling above or below the mean difference is shown inset. (ZIP 221 kb) [file 13047_2017_187_MOESM1_ESM.zip › Supp fig G - SLR3.pdf]

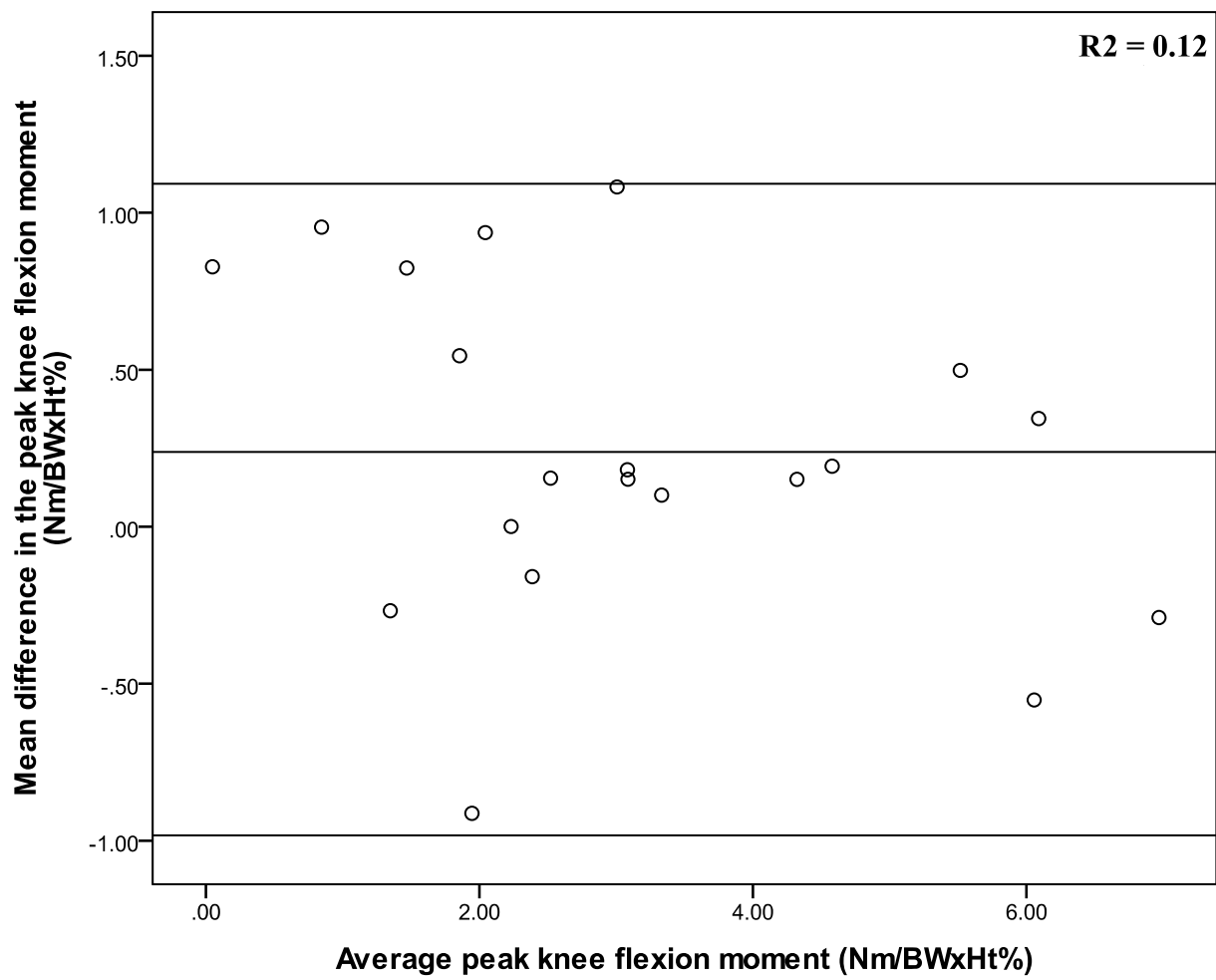

Supplement: Additional file 1: Figure S1. — Description: Multiple figures - supplementary limits of agreement plots for secondary outcome measures. Bland Altman plots and the 95% limits of agreement for the (A) peak knee flexion moment, (B) peak knee flexion in loading response, (C) peak knee extension in stance, (D) peak knee adduction in stance, (E) peak internal rotation in stance, (F) stance duration, (G) stride length, (H) cadence, (I) velocity and (J) base of support. The mean difference between conditions is shown on the Y-axis and the standard deviation of the difference is shown on the X-axis. Regression coefficient of proportional bias in data falling above or below the mean difference is shown inset. (ZIP 221 kb) [file 13047_2017_187_MOESM1_ESM.zip › Supp fig A - KFMR3.pdf]

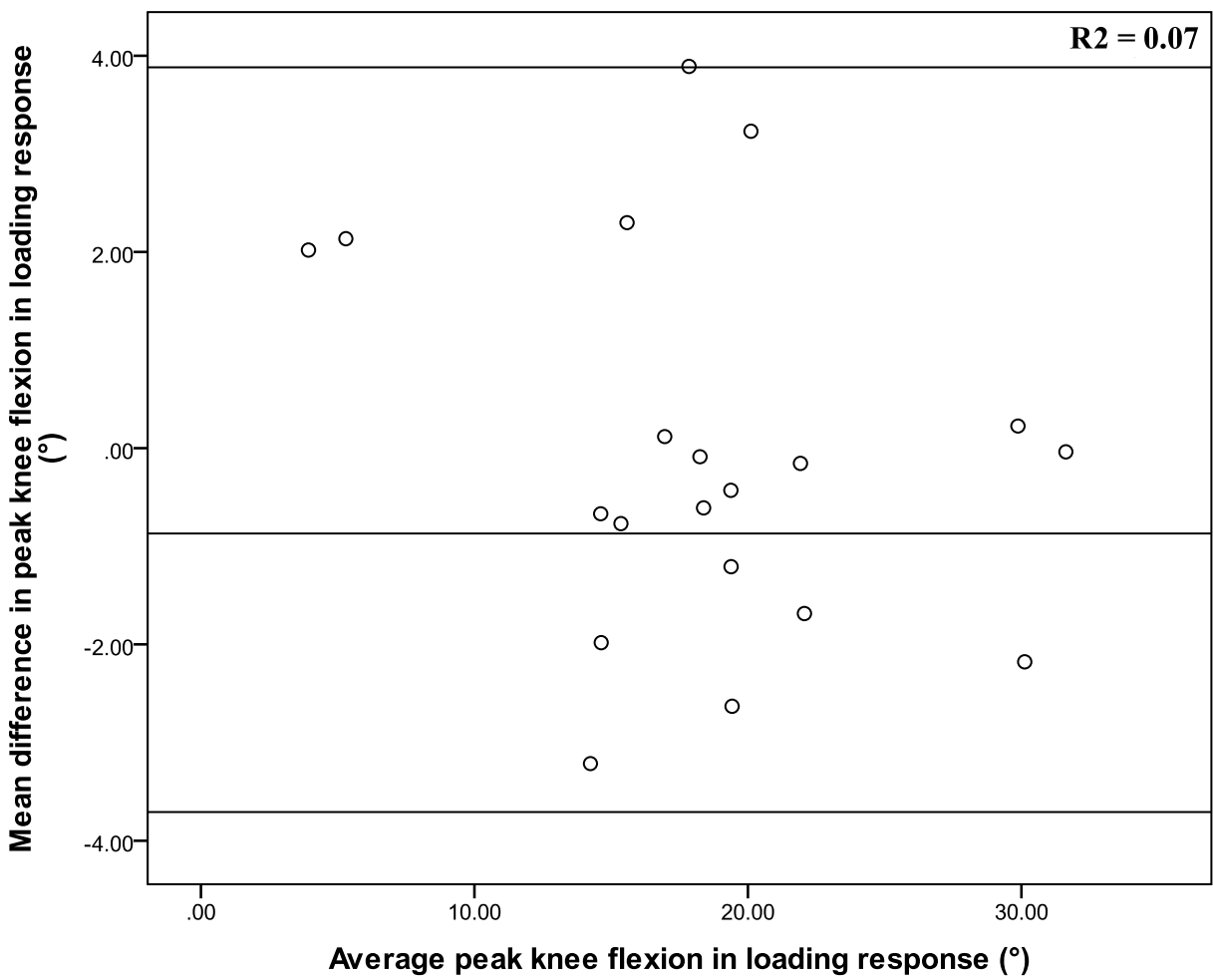

Supplement: Additional file 1: Figure S1. — Description: Multiple figures - supplementary limits of agreement plots for secondary outcome measures. Bland Altman plots and the 95% limits of agreement for the (A) peak knee flexion moment, (B) peak knee flexion in loading response, (C) peak knee extension in stance, (D) peak knee adduction in stance, (E) peak internal rotation in stance, (F) stance duration, (G) stride length, (H) cadence, (I) velocity and (J) base of support. The mean difference between conditions is shown on the Y-axis and the standard deviation of the difference is shown on the X-axis. Regression coefficient of proportional bias in data falling above or below the mean difference is shown inset. (ZIP 221 kb) [file 13047_2017_187_MOESM1_ESM.zip › Supp fig B - KFLRR3.pdf]

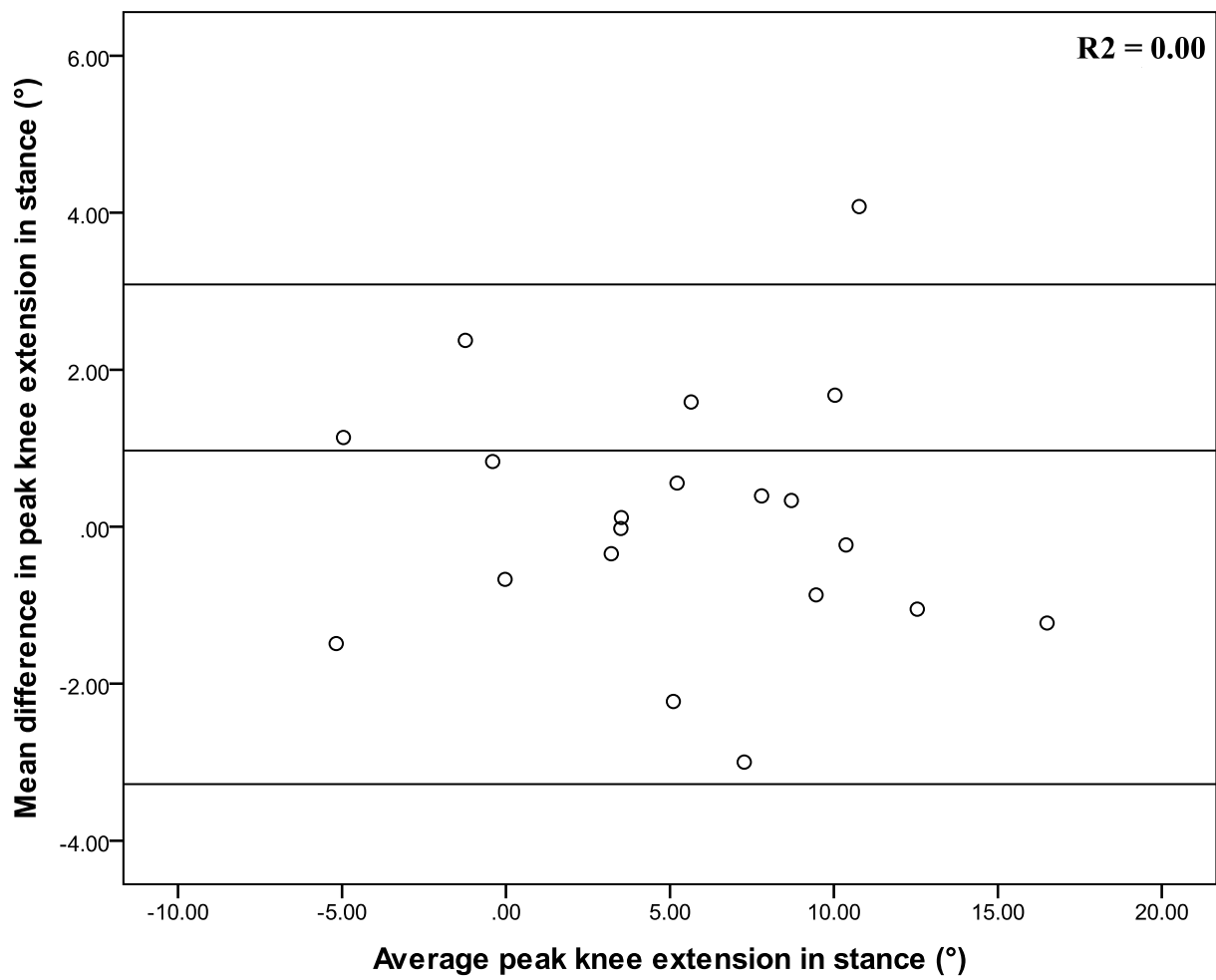

Supplement: Additional file 1: Figure S1. — Description: Multiple figures - supplementary limits of agreement plots for secondary outcome measures. Bland Altman plots and the 95% limits of agreement for the (A) peak knee flexion moment, (B) peak knee flexion in loading response, (C) peak knee extension in stance, (D) peak knee adduction in stance, (E) peak internal rotation in stance, (F) stance duration, (G) stride length, (H) cadence, (I) velocity and (J) base of support. The mean difference between conditions is shown on the Y-axis and the standard deviation of the difference is shown on the X-axis. Regression coefficient of proportional bias in data falling above or below the mean difference is shown inset. (ZIP 221 kb) [file 13047_2017_187_MOESM1_ESM.zip › Supp fig C - KER3.pdf]
